# The Association between Metabolic Syndrome and Epithelial Cell Abnormalities Detected on Pap Smear: A Nationwide Population-Based Study

**DOI:** 10.3390/jcm12082954

**Published:** 2023-04-19

**Authors:** Dayong Lee, Taek Sang Lee

**Affiliations:** Department of Obstetrics and Gynecology, Seoul Metropolitan Government-SNU Boramae Medical Center, Seoul 07061, Republic of Korea; wabhappy@gmail.com

**Keywords:** metabolic syndrome, cervical cytology screening, cervical intraepithelial neoplasia

## Abstract

Several epidemiologic studies have suggested the correlation between metabolic syndrome (MetS) and cervical cancer. The identification of epithelial cell abnormalities through cervical cytology implies lesions that may lead to cervical cancer in the long term, making screening a crucial measure for its prevention. We conducted a case-control study using data from the National Health Screening Programs under the Health Insurance System of South Korea between 2009 and 2017. Among women who underwent a Pap smear during this period, 8,606,394 tests reported negative results for epithelial cell abnormalities (controls, 93.7%), while 580,012 tests reported epithelial cell abnormalities (cases, 6.3%). Of these, the incidence of MetS was significantly higher in the case group, with 21.7% of cases and 18.4% of controls meeting the MetS criteria with *p*-Value of less than 0.0001; however, the effect size was small with odds ratio of 1.23. Logistic regression analysis revealed increased odds of epithelial cell abnormalities in women with MetS after adjusting for associated risk factors (AOR 1.202, 95% CI 1.195–1.210, *p* < 0.0001). These findings indicate that women with MetS have an elevated risk of developing epithelial cell abnormalities, reinforcing the importance of regular Pap smear screening to prevent cervical cancer progression in this population.

## 1. Introduction

Cervical cancer is the fourth most common gynecological cancer globally. About 604,000 new cases and 342,000 deaths were reported in 2020, and most of them are from low- and middle-income countries [1]. In Korea, according to the statistics of the Health Insurance Review and Assessment Service, over 50,000 patients have utilized hospital services for cervical cancer every year from 2016 to 2020. The proportion of patients in their 20s and 30s increased in 2020 compared to 2016. The number of patients in their 20s increased by about 47%, from 2606 in 2016 to 3836 in 2020, while the number of patients in their 30s increased by about 16.7% from 11,966 in 2016 to 13,970 in 2020 [2,3]. Therefore, it is important to emphasize the regular screening of cervical cancer from an early age [4]. Cervical cancer is a preventable malignancy, and it takes a long time for precancerous lesions to develop into invasive cancer. These lesions can be detected through cervical cytology screening, commonly known as a Pap smear. Early detection of these precancerous lesions allows for effective treatment and monitoring for recurrence.

Initiation of cervical dysplasia and carcinogenesis is caused by specific types of human papillomavirus (HPV) infection [5]. The progression from HPV infection to invasive cancer can take several decades [6]. In addition to the causal relationship between HPV and cervical cancer, various acquired and environmental factors are proven to be involved in persistent HPV infection and carcinogenesis. Metabolic syndrome has been proposed as one such factor.

Metabolic syndrome (MetS) is a group of risk factors that can increase the likelihood of developing cardiovascular disease and type 2 diabetes. These risk factors include central obesity, hypertension, dyslipidemia, and hyperglycemia [7]. MetS can also cause chronic inflammation and oxidative stress, which are associated with the development of cancer [8]. Growing epidemiological evidence indicates that MetS is associated with several common cancers, including colon cancer, gastric cancer, esophageal cancer, pancreatic cancer, renal cancer, and liver cancer, as well as cancer-related morbidity and mortality [9]. MetS has also been shown to be correlated with various gynecological cancers [10], and its relationship with cervical cancer has been suggested by multinational studies and population-based database studies [11,12]. However, studies that have analyzed such associations are still limited, and further analyses are necessary. Moreover, given that cervical cancer can be screened for precancerous lesions, it is plausible to assume that women with MetS may also have an increased risk of precancerous cervical lesions. Nevertheless, there is currently no concrete evidence that confirms this assumption.

The purpose of this study is to investigate the association between MetS and abnormal epithelial cell abnormalities detected by a Pap smear, which are precursors of cervical cancer. The study uses population-based national healthcare data from the general health screening program implemented every two years by the National Health Insurance Service (NHIS) in the Republic of Korea. The NHIS health checkup program includes questionnaires on health status and behavior, body measurements, and basic serum and urine tests, as well as a Pap smear test for cervical cancer screening for every woman. The analysis aims to determine whether there is a correlation between MetS and abnormal epithelial cell abnormalities, and to provide insights into the importance of regular Pap smear screenings in populations with MetS to prevent cervical cancer.

## 2. Materials and Methods

### 2.1. Study Design and Database Population

We conducted a case-control study utilizing data from the National Health Insurance Service (NHIS) health checkup program. The NHIS is a non-profit organization established to provide universal health insurance coverage in Korea, and all Korean citizens are required to enroll in the NHIS. The NHIS health checkup program, which is available biannually to all insured Korean citizens [13], includes general health checkups, cancer screenings, and lifetime transition-period health checkups. Anthropometric measurements such as body mass index (BMI), waist circumference, and blood pressure are measured by trained examiners. Starting from 2009, waist circumference has been measured at the umbilicus level by trained nurses or experts. Laboratory tests including fasting blood glucose, total cholesterol, triglycerides, high-density lipoprotein cholesterol, low-density lipoprotein cholesterol, and serum creatinine levels are also included in the health checkup program. Cervical cancer screening using a Pap smear was previously conducted for women aged 30 and above in the cancer program. Since 2016, the Korean government has started providing cervical cancer screening for women aged 20 and above. Self-reported questionnaires are collected to gather information on general health behaviors such as smoking and alcohol consumption. The smoking status was categorized as never-smoker, ex-smoker, or current smoker. Alcohol intake was recorded as a binary variable of yes or no. The hospitals providing these health checkups are certified by the NHIS, and the Korean Association of Quality Assurance for Clinical Laboratories ensures the quality of the laboratory tests.

The study population consists of female individuals who received NHIS health checkups between 2009 and 2017. The NHIS source data was obtained from the Health Insurance Review and Assessment (HIRA) service. Data were collected from a randomly selected sample of 10 million women who underwent NHIS health checkups annually. Women who had undergone a Pap smear screening were selected from the data, and those who were previously diagnosed with cervical cancer were excluded from the analysis based on the survey questionnaire.

The Pap smear results obtained from the NHIS health checkup were categorized into five groups: (1) no abnormal findings, (2) inflammatory or infectious diseases, (3) epithelial cell abnormalities, (4) suspected cervical cancer, and (5) other findings. Women who did not have any epithelial cell abnormalities (including those with no abnormal findings or inflammatory/infectious findings) were categorized as the control group, while women with epithelial cell abnormalities (including those with epithelial cell abnormalities or suspected cervical cancer) were categorized as the case group. In the cervical cancer screening guidelines published by the Ministry of Health and Welfare in the Republic of Korea, it includes both squamous cell abnormalities and glandular cell abnormalities. Within squamous cell abnormalities, atypical squamous cells, low-grade squamous intraepithelial lesion, high-grade squamous intraepithelial lesion, and invasive squamous cell carcinoma are included. Glandular cell abnormalities consist of atypical glandular cells, glandular intraepithelial lesion, and invasive glandular carcinoma. However, since the purpose of this study was to examine epithelial cell abnormalities as a screening tool, these abnormalities were not differentiated in detail. This approach enabled a comparison of cases with epithelial cell abnormalities and negative cases resulting from cervical cancer screening tests.

Metabolic syndrome (MetS) was defined as the presence of three or more of the following conditions: (1) waist circumference ≥ 85 cm as the cut-off value in Korean women [14]; (2) systolic blood pressure ≥ 130 mmHg or diastolic blood pressure ≥ 85 mmHg, or the use of antihypertensive drugs after being diagnosed with hypertension; (3) triglycerides ≥ 150 mg/dL or the use of medication for dyslipidemia; (4) HDL cholesterol level ≤ 50 mg/dL; and (5) fasting serum glucose level ≥ 100 mg/dL, or the use of anti-diabetic medications after being diagnosed with diabetes.

### 2.2. Statistical Analysis

The data were analyzed using SAS software version 9.4 (SAS Institute; Cary, NC, USA). Continuous variables were presented as mean ± SD, while categorical variables were presented as frequency (percentage). Student’s t-test was used to compare means between the two groups, and the Wald chi-square test was used to compare proportions. Due to the large sample size in this analysis, there is a high likelihood of a significant difference in the proportions between the two groups. Therefore, odds ratio (case group odds/control group odds) was calculated to analyze the effect size [15].

Logistic regression analysis was used to calculate the associations between MetS or its components and epithelial cell abnormalities, with or without adjusting for covariates [12]. A *p* Value of less than 0.05 was considered statistically significant.

## 3. Results

The distributions of demographics, MetS component, and covariates as the results of epithelial cell abnormalities on Pap smear are presented in Table 1. The data show that women with epithelial cell abnormalities were older than the control group. When each component of MetS was analyzed, the group with epithelial cell abnormalities had significantly higher ratios of all five individual components compared to the group without abnormalities. The proportion of women meeting the criteria for MetS was significantly higher in the case group (21.7%) than in the control group (18.4%) (*p* < 0.0001). According to the questionnaire data, the rate of alcohol intake was significantly higher in the group without epithelial cell abnormalities (11.0%) than in the group with abnormalities (9.1%). Moreover, the proportion of women who were current or ex-smokers was significantly higher in the group without epithelial cell abnormalities (5.7%) compared to the group with abnormalities (4.9%). The odds ratio was highest in hypertension at 1.31, followed by MetS at 1.23. However, all were less than 1.5, indicating small effect sizes for all variables [15].

Table 2 summarizes the results of the univariate logistic regression analysis. In univariate analysis, women with MetS had higher odds of developing epithelial cell abnormalities (OR = 1.23, 95% CI 1.22–1.24, *p* < 0.0001). When each component of MetS was individually analyzed, the odds of having epithelial cell abnormalities significantly increased for every component. Univariate logistic regression analysis was performed based on the number of components of MetS satisfied, and the odds ratio increased from 1.14 to 1.36 for up to three components. However, when three or more components were present, the odds ratio did not increase further. After adjusting for age, MetS, drinking, and smoking status through multivariate logistic regression analysis, the odds of epithelial cell abnormalities in women with MetS significantly increased to 1.20 (95% CI 1.20–1.21, *p* < 0.0001), as presented in Table 3.

## 4. Discussion

This study conducted an analysis of population-based data obtained from a nationwide general health screening program to investigate the relationship between MetS and epithelial cell abnormalities detected through Pap smear screening in women. The findings revealed that the incidence of MetS was higher in women with abnormal epithelial cell abnormalities compared to those with a negative result for such abnormalities. However, the effect sizes for all variables were small in the analysis using odds ratio. After adjusting for associated risk factors including age, the odds of epithelial cell abnormalities in women with MetS were found to be significantly increased. The study suggests that MetS may impact the host’s ability to combat HPV and may exacerbate abnormal epithelial cell changes caused by persistent HPV infection, thereby increasing the long-term risk of cervical cancer. This is the first population-based study to analyze the association between MetS and abnormal cervical epithelial cell abnormalities.

Previous large population-based epidemiological studies have suggested a correlation between MetS and the incidence of cervical cancer. Penaranda et al. reported this correlation through the outcome of a case-control study using the United States population survey data. Their logistic regression analysis reported that women with MetS had 1.91-times higher odds of cervical cancer in unadjusted (95% CI 1.06–3.42, *p* = 0.0309) and 1.82-times higher odds in covariates adjusted calculations including multiple lifetime sexual partners, higher parity, hormonal contraceptive use, and history of smoking (95% CI 1.02–3.26, *p* = 0.0428) [12]. In this study, each separate component of the MetS was not significantly correlated with cervical cancer. When comparing these results with the outcomes of the current study, it can be noted that each component of MetS can cause epithelial cell abnormalities, but each component alone is not sufficient to act as a causal risk factor for cervical cancer. This difference suggests that MetS is not simply the sum of each component, but an individual entity of disease that can affect long-term health outcomes. Ulmer et al. conducted a prospective cohort study over a mean follow-up duration of 11 years. They observed a population of 288,834 individuals and found 425 cases of invasive cervical cancer. They estimated the hazard ratio using Cox proportional hazards regression models for quintiles and standardized z-scores (with a mean of 0 and a standard deviation of 1) of each MetS component. Using this method, they constructed a MetS score to evaluate the association between MetS and cervical cancer risk. The MetS score was found to be associated with a 26% increased risk of cervical cancer after adjustment for confounding factors. Upon analysis of each component of MetS, it was found that only elevated triglyceride levels were significantly associated with the occurrence of cervical cancer. These findings suggest that women with MetS have a higher risk of cervical cancer compared to those without the syndrome.

Recent studies have suggested that MetS may influence HPV infection in women. For example, Huang et al. reported that women with MetS have an increased risk of HPV infection (RR 1.25, 95% CI 1.09–1.46) [16]. In studies analyzing the components of MetS, obesity was found to be a major variable mediating the association between MetS and incident HPV infection. Among obese individuals, hypertriglyceridemia was found to be significantly associated with an increased risk of HPV infection. Molokwu et al. conducted a study that included a male cohort, which could potentially serve as a mediator of HPV infection in females. They found that MetS was significantly associated with an increased risk of HPV with 6, 11, 16, or 18 infections in the total cohort (RR 1.24, 95% CI 1.03–1.48) as well as in the female cohort (RR 1.26, 95% CI 1.02–1.56) [17]. Upon analyzing the individual components of MetS in both male and female participants, it was found that increased waist circumference, low HDL cholesterol, increased blood pressure, and age were significantly associated with HPV infection. The findings of the studies discussed suggest that individual components of MetS, such as obesity, dyslipidemia, and hypertension, are associated with HPV infection. These results are consistent with the findings of the current study, which indicates that all components of MetS are associated with abnormal epithelial cell results of the uterine cervix. Furthermore, this study also observed synergistic effects of the components of MetS. As the number of MetS components increased from one to three, the odds ratio of abnormal epithelial cell results of the uterine cervix continuously increased. However, this increase in risk was no longer observed in cases where there were three or more MetS components. This finding is in line with the diagnostic criteria for MetS, which require three or more criteria to be met.

Persistent HPV infection is known to trigger cervical dysplasia, which can progress to cervical cancer if left untreated for several decades. If MetS contributes to persistent HPV infection, it may also increase the prevalence of cervical dysplasia in women with MetS [6]. However, there is currently no epidemiological data available on this topic. Therefore, if MetS increases the risk of HPV infection and is associated with the development of cervical cancer, there must be an intermediate process involving epithelial cell abnormalities. This study provides population-based evidence supporting this association.

Studies have shown that women with persistent HPV infection have increased levels of adipokines, cytokines, and inflammatory markers. These findings suggest that components of MetS may contribute to immune compromise and promote HPV-related carcinogenesis. In this study, univariate logistic regression analysis was performed for each component of MetS, and it was found that the odds of central obesity, hypertension, dyslipidemia, and hyperglycemia were all significantly increased. These components have been shown to contribute to immune compromise, increasing susceptibility to viral infections, and potentially lead to carcinogenesis. Adipose tissue plays a role as an endocrine organ by regulating hormonal environments and circulating cytokines such as sex steroids, leptin, adipokines, tumor necrosis factor (TNF)-alpha, and plasminogen activator inhibitor-1 [18]. Dysregulation of these factors and the inability to efficiently store excess free fatty acids in adipose cells contribute to chronic inflammation and carcinogenesis [19]. In MetS, hyperinsulinemia caused by insulin resistance and subsequent hyperglycemia can trigger carcinogenesis by increasing the circulating levels of free insulin-like growth factor (IGF)-1 [20]. Impaired glucose metabolism and hyperglycemia can promote malignant cell proliferation. Glucose transport proteins, including glucose transporter-1 (GLUT-1) in malignant cells, are known to support the high glucose demands for rapid tumor growth [21]. High glucose concentrations can promote the invasion and metastasis of malignant cells through the stimulation of epithelial-mesenchymal transition (EMT), which is a critical pathway for acquiring migration, invasion, and pluripotent stem cell-like phenotypes [22]. Hypertriglyceridemia can stimulate cell proliferation and promote anti-apoptotic capacity by activating the synthesis of reactive oxygen species (ROS) and inducing DNA damage through oxidative stress [9].

The current study found that women who smoke or drink had a reduced incidence of epithelial cell abnormalities, when considering habitual risk factors. However, it is important to note that the case group and control group were divided based on the presence or absence of epithelial cell abnormalities, respectively. Inflammatory or infectious diseases without epithelial cell abnormalities were included in the control group. Smoking and drinking habits are often associated with individual lifestyles, including sexual behavior, which can increase the risk of inflammatory or infectious diseases in women, as well as HPV infection. According to some studies, individuals who consume alcohol frequently may have a higher prevalence of sexually transmitted infections [23]. The control group in this study also included cases of sexually transmitted infections, including cervicitis. Since the prevalence of these diseases is more common than the occurrence of epithelial cell abnormalities, the study results should be interpreted with this context.

The limitation of this study is that there are no data available for several potential confounding factors, including sexual behavior (such as the number of sexual partners or age of first sexual intercourse), parity, contraceptive methods, and socioeconomic status. This information was not included in the NHIS general health screenings questionnaire, so it was not possible to include these variables in the analysis. As the number of lifetime sexual partners is an established risk factor for cervical cancer, it is important to note that these potential confounders will be further analyzed together.

In conclusion, our analysis of national-wide population-based health checkup data revealed an elevated risk of epithelial cell abnormalities detected through a Pap smear screening in women with MetS. While each component of MetS was found to increase the likelihood of epithelial cell abnormalities, we observed a synergistic effect when three or more components were present. MetS has been shown to increase the risk of developing cervical cancer, making it all the more important for women with MetS to undergo regular Pap smear screenings. Pap smears are a highly effective method for preventing and detecting cervical cancer in its early stages. Therefore, it is crucial to emphasize the importance of regular Pap smear screening for women with MetS.

## Figures and Tables

**Table 1:** Characteristics of populations stratified by the result of epithelial cell abnormalities on a Pap smear.

Characteristics	Cases (*n* = 580,012)	Controls (*n* = 8,606,394)	*p* Value	OR
Age (mean ± SD, years)	54.3 ± 10.4	50.9 ± 9.9	<0.0001	
Age ≥ 40 years	94.2	91.9	<0.0001	1.43
Components of metabolic syndrome				
Waist circumference ≥ 85 cm	19.5	18.1	<0.0001	1.10
Hypertension	40.5	34.2	<0.0001	1.31
Hypertriglyceridemia	20.3	18.5	<0.0001	1.12
Low HDL cholesterol	34.1	31.2	<0.0001	1.14
Hyperglycemia	31.3	28.8	<0.0001	1.13
Metabolic syndrome (≥3 components)	21.7	18.4	<0.0001	1.23
Alcohol intake	9.1	11.0	<0.0001	0.81
Smoking status			<0.0001	0.85 *
Never-smoker	95.1	94.3		
Ex-smoker	2.0	2.2		
Current smoker	2.9	3.5		

SD, standard deviation; HDL, high density lipoprotein; OR, odds ratio. * OR for ever-smoker (ex-smoker + current smoker).

**Table 2:** Univariate logistic regression analysis for the presence of metabolic syndrome and epithelial cell abnormalities on a Pap smear.

	Univariate Model		
	OR	95% CI	*p* Value
Metabolic syndrome (≥3 components)	1.23	1.22, 1.24	<0.0001
5 components vs. no components	1.37	1.34, 1.39	<0.0001
4 components vs. no components	1.40	1.39, 1.42	<0.0001
3 components vs. no components	1.36	1.35, 1.37	<0.0001
2 components vs. no components	1.28	1.27, 1.29	<0.0001
1 component vs. no components	1.14	1.13, 1.15	<0.0001
Waist circumference ≥85 cm	1.10	1.09, 1.10	<0.0001
Hypertension	1.31	1.30, 1.32	<0.0001
Hypertriglyceridemia	1.12	1.11, 1.13	<0.0001
Low HDL	1.14	1.13, 1.15	<0.0001
Hyperglycemia	1.13	1.12, 1.13	<0.0001

HDL, high density lipoprotein; OR, odds ratio; CI, confidence interval.

**Table 3:** Multivariate logistic regression analysis for the presence of metabolic syndrome and epithelial cell abnormalities on a Pap smear.

	Multivariate Model		
	AOR	95% CI	*p* Value
Age ≥ 40 years	1.37	1.35, 1.38	
Metabolic syndrome (≥3 components)	1.20	1.20, 1.21	<0.0001
Alcohol intake	0.86	0.85, 0.87	<0.0001
Smoking (Smoker vs. never-smoker)	0.88	0.87, 0.90	<0.0001
(Ex-smoker vs. never-smoker)	0.93	0.91, 0.95	<0.0001

AOR, adjusted odds ratio; CI, confidence interval.

## Data Availability

This study analyzed data provided by the National Health Insurance Sharing Service (NHISS) of Republic of Korea. The export or public disclosure of raw data is prohibited by the regulations of the institution.

## References

[1] Sung H., Ferlay J., Siegel R.L., Laversanne M., Soerjomataram I., Jemal A., Bray F. (2021). Global cancer statistics 2020: GLOBOCAN estimates of incidence and mortality worldwide for 36 cancers in 185 countries. CA Cancer J. Clin..

[2] Health Insurance Review & Assessment Service National Interest Disease Statistics. http://opendata.hira.or.kr/op/opc/olapMfrnIntrsIlnsInfo.do.

[3] Ha H.I., Chang H.K., Park S.J., Lim J., Won Y.J., Lim M.C. (2021). The incidence and survival of cervical, ovarian, and endometrial cancer in Korea, 1999-2017: Korea Central Cancer Registry. Obstet. Gynecol. Sci..

[4] Ghalavandi S., Heidarnia A., Zarei F., Beiranvand R. (2021). Knowledge, attitude, practice, and self-efficacy of women regarding cervical cancer screening. Obstet. Gynecol. Sci..

[5] Schiffman M., Wentzensen N., Wacholder S., Kinney W., Gage J.C., Castle P.E. (2011). Human papillomavirus testing in the prevention of cervical cancer. J. Natl. Cancer Inst..

[6] Rodríguez A.C., Schiffman M., Herrero R., Hildesheim A., Bratti C., Sherman M.E., Solomon D., Guillén D., Alfaro M., Morales J. (2010). Longitudinal study of human papillomavirus persistence and cervical intraepithelial neoplasia grade 2/3: Critical role of duration of infection. J. Natl. Cancer Inst..

[7] Haffner S.M., Valdez R.A., Hazuda H.P., Mitchell B.D., Morales P.A., Stern M.P. (1992). Prospective analysis of the insulin-resistance syndrome (syndrome X). Diabetes.

[8] Alberti K.G., Eckel R.H., Grundy S.M., Zimmet P.Z., Cleeman J.I., Donato K.A., Fruchart J.C., James W.P., Loria C.M., Smith S.C. (2009). Harmonizing the metabolic syndrome: A joint interim statement of the international diabetes federation task force on epidemiology and prevention; national heart, lung, and blood institute; American heart association; world heart federation; international atherosclerosis society; and international association for the study of obesity. Circulation.

[9] Cowey S., Hardy R.W. (2006). The metabolic syndrome: A high-risk state for cancer?. Am. J. Pathol..

[10] Lee D.Y., Lee T.S. (2020). Associations between metabolic syndrome and gynecologic cancer. Obstet. Gynecol. Sci..

[11] Ulmer H., Bjørge T., Concin H., Lukanova A., Manjer J., Hallmans G., Borena W., Häggström C., Engeland A., Almquist M. (2012). Metabolic risk factors and cervical cancer in the metabolic syndrome and cancer project (Me–Can). Gynecol. Oncol..

[12] Penaranda E.K., Shokar N., Ortiz M. (2013). Relationship between metabolic syndrome and history of cervical cancer among a US national population. Int. Sch. Res. Not..

[13] Song S.O., Jung C.H., Song Y.D., Park C.Y., Kwon H.S., Cha B.S., Park J.Y., Lee K.U., Ko K.S., Lee B.W. (2014). Background and data configuration process of a nationwide population-based study using the korean national health insurance system. Diabetes Metab. J..

[14] Park H.S., Park C.Y., Oh S.W., Yoo H.J. (2008). Prevalence of obesity and metabolic syndrome in Korean adults. Obes. Rev..

[15] Sullican G.M., Feinn R. (2012). Using Effect Size—Or Why the P Value Is Not Enough. J. Grad. Med. Educ..

[16] Huang X., Zhao Q., Yang P., Li Y., Yuan H., Wu L., Chen Z. (2016). Metabolic syndrome and risk of cervical human papillomavirus incident and persistent infection. Medicine.

[17] Molokwu J.C., Penaranda E., Lopez D.S., Dwivedi A., Dodoo C., Shokar N. (2017). Association of Metabolic Syndrome and Human Papillomavirus Infection in Men and Women Residing in the United States. Cancer Epidemiol. Biomark. Prev..

[18] Lee S.W., Cho H.H., Kim M.R., Kwon D.J., Jung E., You Y.O., Kim J.H. (2010). The relationship between serum leptin level and metabolic syndrome in postmenopausal women. Korean J. Obstet. Gynecol..

[19] Van Kruijsdijk R.C., Van Der Wall E., Visseren F.L. (2009). Obesity and Cancer: The Role of Dysfunctional Adipose Tissue Obesity and Cancer. Cancer Epidemiol. Biomark. Prev..

[20] Iyengar N.M., Gucalp A., Dannenberg A.J., Hudis C.A. (2016). Obesity and cancer mechanisms: Tumor microenvironment and inflammation. J. Clin. Oncol..

[21] Macheda M.L., Rogers S., Best J.D. (2005). Molecular and cellular regulation of glucose transporter (GLUT) proteins in cancer. J. Cell Physiol..

[22] Iwatsuki M., Mimori K., Yokobori T., Ishi H., Beppu T., Nakamori S., Baba H., Mori M. (2010). Epithelial–mesenchymal transition in cancer development and its clinical significance. Cancer Sci..

[23] Jung M. (2019). Risk factors of sexually transmitted infections among female sex workers in Republic of Korea. Infect. Dis. Poverty.

